# Trends and comparative analysis of the burden of migraine in China and globally from 1990 to 2021: an analysis based on the 2021 global burden of disease study

**DOI:** 10.3389/fneur.2025.1592224

**Published:** 2025-11-03

**Authors:** Bo Peng, Yuluo Tu, Suifa Hu, Gui Xie

**Affiliations:** Nanchang Hongdu Hospital of Traditional Chinese Medicine, Nanchang, Jiangxi, China

**Keywords:** migraine, incidence, prevalence, disability-adjusted life-years, global burden of disease study 2021

## Abstract

**Background:**

Migraine is a common neurological disorder that has become an increasingly significant public health issue. This study aims to analyze the burden of migraine in China and globally from 1990 to 2021, exploring epidemiological trends and differences, thus providing scientific evidence for migraine prevention and control.

**Methods:**

Based on the 2021 Global Burden of Disease (GBD) study, we assessed migraine burden in China and globally from 1990 to 2021 using indicators including incidence, prevalence, disability-adjusted life years (DALYs), and age-standardized rates. The epidemiological trends were analyzed by calculating the estimated annual percentage change (EAPC). Health inequality analysis was conducted to explore the association between migraine burden and the sociodemographic index (SDI). Decomposition analysis quantified contributions of age structure, population growth, and epidemiological changes to migraine burden. Additionally, the Bayesian Age-Period-Cohort (BAPC) model was applied to predict migraine burden in China and globally over the next 10 years.

**Results:**

Compared with 1990, the number of migraine cases, prevalence, and DALYs in both China and globally significantly increased by 2021, though the global growth rate was considerably higher. Between 1990 and 2021, China experienced greater increases in age-standardized incidence rates (ASIR), age-standardized prevalence rates (ASPR), and age-standardized DALYs rates (ASDR) than the global average. Migraine burden was predominantly concentrated among adolescents and young adults, and females consistently exhibited a higher burden than males. Health inequality analysis revealed increasing disparity across 204 countries and regions, with a concentrated migraine burden in high socio-demographic index (SDI) countries, positively correlated with SDI. Decomposition analysis indicated that population growth was the primary driver of migraine burden changes in both China and globally. BAPC modeling predicted that the age-standardized incidence, prevalence, and DALY rates for migraine will continue to rise in China, whereas these rates are expected to slightly decline globally.

**Conclusions:**

Migraine burden is rising in both China and globally, driven by multiple factors such as age, gender, population growth, and SDI. There is an urgent need for precise interventions to reduce migraine's public health impact.

## Introduction

Migraine, as a highly prevalent and disabling neurological disorder, has emerged as a major public health concern worldwide (1, 2). Existing evidence indicates that migraine not only substantially impairs individual quality of life but also contributes to elevated healthcare expenditures and productivity loss (3). In the Asia–Pacific region, the combination of a large population base and heterogeneous socioeconomic development results in a complex and diverse disease burden (4, 5). Although the Global Burden of Disease (GBD) study (6) has provided valuable epidemiological data on migraine, current research generally lacks systematic comparisons across regions and sociodemographic contexts. This gap limits the precision of policymaking and the equitable allocation of healthcare resources for migraine prevention and management.

In recent years, research on the global burden of migraine has been increasing; however, most studies have primarily focused on overall epidemiological trends at the global or regional level, with limited systematic comparisons between China and the rest of the world (7–9). Moreover, the majority of existing literature relies on data from 2019 or earlier, which fails to capture the indirect effects of the COVID-19 pandemic on the burden of neurological disorders (10, 11). Given the profound impact of the pandemic on healthcare resource allocation, public health priorities, and population mental health, the burden of migraine may have been exacerbated across different regions and populations (12). In the post-pandemic era, substantial changes in healthcare accessibility, the effectiveness of health interventions, and lifestyle behaviors are likely to have reshaped migraine epidemiology and disease burden (13, 14). Therefore, a comprehensive analysis of the differences in migraine burden between China and the global population using the most up-to-date post-pandemic data has become an urgent research priority.

This study, based on data from the GBD 2021 database (15), systematically evaluated temporal trends in key epidemiological indicators of migraine—including incidence, prevalence, and disability-adjusted life years (DALYs)—from 1990 to 2021 at both global and Chinese levels. From the perspective of health inequality, we further examined differences in disease burden across countries with varying levels of socioeconomic development and employed decomposition analysis to disentangle the relative contributions of population growth, demographic shifts, and epidemiological changes. In addition, we developed a Bayesian age–period–cohort (BAPC) model to project the future burden of migraine in China and globally through 2031. Distinct from previous studies, this research integrates the most recent post-pandemic data and provides a direct comparison between China and the global population, thereby revealing the heterogeneous characteristics of migraine burden. These findings are expected to provide robust scientific evidence for optimizing global public health strategies, improving resource allocation, and guiding precision interventions for migraine prevention and management.

## Methods

### Data source

The data used in this study is from the 2021 GBD database, which is widely recognized as one of the largest and most authoritative health data repositories in the world. GBD 2021, led by the Global Health Research Collaboration Network, is an epidemiological study covering 204 countries and regions from 1990 to 2021, assessing 371 diseases, injuries, and 88 risk factors. The study aims to quantify the disease and risk burden at various levels and provide scientific evidence for the formulation of public health policies. The data in the GBD database comes from a wide range of sources, including population surveys, national censuses, disease surveillance systems, and death registration reports. All data is categorized by region, year, gender, and age, and is cleaned, calibrated, and adjusted through Bayesian hierarchical models and the Cause of Death Ensemble Model to eliminate data heterogeneity and potential biases, ensuring high-accuracy estimates. Additionally, the GBD uses the Population Attributable Fraction method to quantify the contribution of specific risk factors to the disease burden (15). This study extracted migraine burden data for China and globally from 1990 to 2021, primarily including indicators such as incidence, prevalence, DALYs, ASIR, ASPR, and ASDR, and provided corresponding 95% uncertainty intervals (UI) to assess the reliability of the statistical results. All data was retrieved through the Global Health Data Exchange platform (website: https://ghdx.healthdata.org/gbd-2021). Data extraction strictly followed the standardized procedures of GBD to ensure consistency in time span, geographical scope, and data quality. This study used publicly available anonymous data in accordance with the Guidelines for Accurate and Transparent Health Estimates Reporting, and therefore, ethical approval was not required (16).

### Definitions

In the GBD 2021 classification system, migraine is categorized as a Level 3 disease under neurological disorders (Level 2) and non-communicable diseases (Level 1) (17). As one of the most common chronic neurological diseases worldwide, migraine holds a significant place among neurological disorders (18). The pathophysiology of migraine is complex, involving abnormal neurovascular responses, and is known to be closely related to genetic susceptibility, neurotransmitter imbalances, and external environmental factors (such as stress, diet, and climate changes) (19). In the International Classification of Diseases (ICD) system, the code for migraine is 346.0-346.9 in ICD-9 and G43.0-G43.9 in ICD-10 (20).

Incidence refers to the number of new cases of a disease occurring in a specific population over a defined period, reflecting the frequency and speed of disease onset. Prevalence, on the other hand, indicates the proportion of all existing cases—both new and pre-existing—within a specific population at a given point in time, measuring the overall extent of the disease and serving as a key indicator for assessing its long-term impact. DALYs are a composite measure of health loss, calculated as the sum of years of life lost due to premature mortality and years lived with disability, thus capturing both fatal and non-fatal health burdens.

ASIR, ASPR, and ASDR are age-standardized metrics that adjust data according to a global standard population, eliminating biases introduced by differences in age structures and enhancing comparability across regions or populations. ASIR reflects variations in disease incidence, enabling precise assessment of epidemiological trends. ASPR evaluates the disease burden within a specific population at a given time, ensuring accurate comparisons of prevalence. ASDR adjusts DALYs for age structure differences, allowing fair comparisons of disease burden across populations or regions and providing a standardized framework for global disease burden assessment.

The SDI is a composite metric used to assess the socioeconomic development level of countries or regions. It is based on three key indicators: per capita income, mean educational attainment among individuals aged 15 years and older, and the total fertility rate among women aged 15–49 years. The SDI ranges from 0 to 1, with higher scores indicating higher levels of socioeconomic development. Based on SDI values, the GBD study classifies 204 countries and territories into five categories: high SDI, high-middle SDI, middle SDI, low-middle SDI, and low SDI (21). This classification facilitates the analysis of the impact of socioeconomic development on disease burden.

The Slope Index of Inequality (SII) and the Concentration Index of Inequality (CII) are key metrics for quantifying health inequalities. The SII measures the absolute difference in health burden between socioeconomic groups, with SII > 0 indicating a higher burden among higher socioeconomic groups and SII < 0 indicating a higher burden among lower socioeconomic groups. The CII assesses the relative concentration of disease burden across the socioeconomic spectrum, where CII > 0 signifies a greater burden in higher socioeconomic groups, and CII < 0 indicates a greater burden in lower socioeconomic groups (22).

### Statistical analysis

This study utilized the 2021 GBD database to systematically assess the epidemiological burden and temporal trends of migraine in China and globally from 1990 to 2021, employing key metrics including incidence, prevalence, DALYs, and age-standardized rates. To mitigate potential bias from differences in age structure, UI-derived disease burden data were used to ensure the accuracy and reliability of the results. Temporal trends were quantified using the EAPC and its 95% confidence interval (CI) calculated via a natural logarithmic regression model. To evaluate health inequalities, the association between migraine burden and the SDI was analyzed by stratifying countries into five SDI quintiles, comparing disease burden across different socioeconomic levels, and quantifying the strength of association using scatter plots and Spearman rank correlation analysis. Decomposition analysis was conducted to estimate the relative contributions of population growth, changes in age structure, and epidemiological shifts to the observed trends in disease burden. Future projections were generated using a BAPC model, incorporating historical data and population forecasts to estimate migraine burden from 2022 to 2031. All analyses and visualizations were performed using R software (version 4.2.2) and the JD_GBDR tool (version 2.37; Jingding Medical Technology Co., Ltd.), with statistical significance defined as *p* < 0.05.

## Results

### Trend analysis of migraine incidence in China and globally

**Figure 1:**
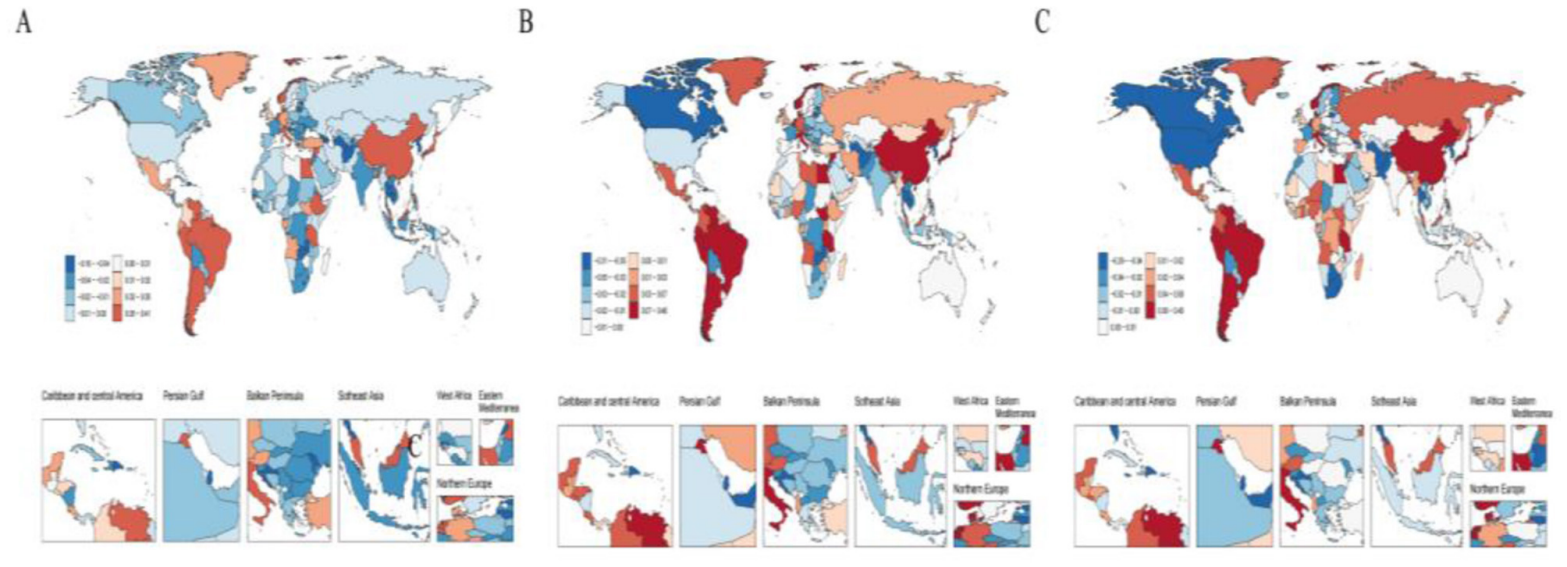
EAPC in ASIR, ASPR, and ASDR of migraine across 204 countries and territories from 1990 to 2021. **(A)** EAPC of migraine ASIR. **(B)** EAPC of migraine ASPR. **(C)** EAPC of migraine ASDR.

In 2021, the number of migraine cases in China was 13,047,220.65 (95% UI: 11,597,731.48–14,698,852.13), representing a 13.28% increase compared with 1990. Among these, the number of male cases increased from 4,341,951.31 in 1990 to 4,965,958.22 in 2021, a growth of 14.37%, while female cases rose from 7,176,146.24 to 8,081,262.43, a 12.61% increase. The ASIR of migraine in China increased from 917.35 per 100,000 (95% UI: 808.35–1,036.95) in 1990 to 975.61 per 100,000 (95% UI: 862.32–1,102.06) in 2021, corresponding to an EAPC of 0.24 (95% CI: 0.20–0.28). The ASIR growth in males (EAPC: 0.28) was slightly higher than in females (EAPC: 0.23) (Table 1). By comparison, the global number of migraine cases in 2021 was 90,183,386.87 (95% UI: 78,857,600.46–101,838,162.48), reflecting a 42.03% increase since 1990. Globally, male cases rose from 23,992,423.67 in 1990 to 34,753,709.72 in 2021, an increase of 44.85%, while female cases increased from 39,504,167.15 to 55,429,677.15, representing a 40.31% rise. The global ASIR also showed an upward trend, with an EAPC of 0.07 (95% CI: 0.05–0.08); the increase was more pronounced in males (EAPC: 0.13) than in females (EAPC: 0.03) (Table 1, Figure 1).

**Table 1:** Trends in the incidence, prevalence, and DALYs of migraine in China and globally from 1990 to 2021.

**Location**	**Measure**	**1990**		**2021**		**1990–2021**
		**Number (95% UI)**	**ASR, per 100,000 (95% UI)**	**Number (95% UI)**	**ASR, per 100,000 (95% UI)**	**EAPC (95% CI)**
**China**						
	**Both**					
	Incidence	11,518,097.55 (10,091,942.15, 13,156,841.89)	917.35 (808.35, 1,036.95)	13,047,220.65 (11,597,731.48, 14,698,852.13)	975.61 (862.32, 1,102.06)	0.24 (0.20, 0.28)
	Prevalence	133,474,536.55 (114,199,443.66, 153,482,597.68)	10,948.52 (9,428.76, 12,586.13)	184,752,280.11 (160,836,524.72, 213,633,958.29	11,777.51 (10,137.56, 13,538.56)	0.28 (0.24, 0.33)
	DALYs	5,028,787.46 (767,668.49, 11,262,271.45)	412.97 (66.16, 911.02)	6,988,198.60 (1,133,318.67, 15,186,289.26)	443.65 (66.93, 971.68)	0.27 (0.23, 0.32)
	**Male**					
	Incidence	4,341,951.31 (3,779,869.64, 4,957,833.48)	668.04 (585.69, 761.40)	4,965,958.22 (4,347,456.23, 5,638,304.94)	722.18 (629.13, 825.03)	0.28 (0.24, 0.33)
	Prevalence	50,887,361.36 (43,387,428.18, 59,129,685.48)	8,077.76 (6,940.56, 9,333.35)	70,036,222.95 (60,652,046.08, 81,324,228.20)	8,781.96 (7,497.40, 10,129.33)	0.30 (0.25, 0.34)
	DALYs	1,987,263.27 (394,504.68, 4,365,126.26)	316.56 (65.91, 692.31)	2,745,320.72 (581,676.86, 5,851,443.22)	341.31 (67.72, 739.24)	0.27 (0.22, 0.31)
	**Female**					
	Incidence	7,176,146.24 (6,275,054.92, 8,198,406.53)	1,183.75 (1,035.22, 1,331.07)	8,081,262.43 (7,209,928.84, 9,102,490.19)	1,252.68 (1,112.53, 1,403.87)	0.23 (0.19, 0.26)
	Prevalence	82,587,175.18 (7,0478,899.45, 94,673,001.54)	13,992.18 (12,040.43, 16,050.32)	114,716,057.16 (98,856,230.57, 132,076,637.56)	14,959.04 (12,857.78, 17,190.35)	0.27 (0.22, 0.31)
	DALYs	3,041,524.19 (360,236.18, 6,866,451.03)	515.35 (64.62, 1,157.38)	4,242,877.87 (548,619.60, 9,335,915.43)	552.65 (64.68, 1,221.52)	0.27 (0.22, 0.32)
**Global**						
	**Both**					
	Incidence	63,496,590.82 (55,194,751.45, 72,208,003.40)	1,136.90 (995.14, 1,287.76)	90,183,386.87 (78,857,600.46, 101,838,162.48)	1,153.20 (1,006.07, 1,304.49)	0.07 (0.05, 0.08)
	Prevalence	732,564,462.68 (624,559,243.92, 84,7058,436.26)	14,027.65 (12,063.37, 16,078.07)	1,158,432,823.82 (995,861,966.38, 1,331,312,506.13)	14,246.55 (12,194.12, 16,378.70)	0.06 (0.05, 0.08)
	DALYs	27,412,196.29 (4,076,605.01, 60,325,805.84)	526.76 (83.36, 1,145.92)	43,378,889.81 (6,732,642.21, 95,079,454.09)	532.70 (80.57, 1,167.71)	0.05 (0.04, 0.07)
	**Male**					
	Incidence	23,992,423.67 (20,793,827.28, 27,423,189.03)	846.91 (735.94, 963.38)	34,753,709.72 (30,126,608.19, 39,711,731.23)	876.55 (758.19, 1,002.54)	0.13 (0.11, 0.14)
	Prevalence	269,667,853.33 (227,787,523.42, 312,882,000.72)	10,229.25 (8,743.81, 11,822.93)	433,190,631.84 (368,788,829.32, 501,602,102.35)	10,624.20 (9,039.46, 12,297.27)	0.13 (0.11, 0.15)
	DALYs	10,232,568.59 (1,675,317.81, 22,688,369.40)	389.98 (68.44, 844.94)	16,494,946.45 (2,802,071.63, 35,551,602.46)	403.88 (67.39, 872.77)	0.13 (0.11, 0.14)
	**Female**					
	Incidence	39,504,167.15 (34,479,511.02, 44,792,382.28)	1,435.60 (1,257.48, 1,617.92)	55,429,677.15 (48,751,162.78, 62,552,034.25)	1,438.90 (1,261.41, 1,620.05)	0.03 (0.02, 0.04)
	Prevalence	462896609.35 (396,950,116.14, 532,406,329.56)	17864.62 (15,368.19, 20,418.65)	725242191.97 (62,7617,457.67, 830,850,946.92)	17902.60 (15,445.99, 20,487.01)	0.02 (0.01, 0.03)
	DALYs	17,179,627.70 (2,400,079.70, 37,859,718.47)	664.92 (98.10, 1453.28)	26,883,943.36 (3,930,570.58, 58,848,028.71)	662.76 (93.66, 1,450.83)	0.01 (−0.00, 0.02)

ASR, age-standardized rates; DALYs, disability-adjusted life years; UI, uncertainty interval; CI, confidence Interval; EAPC, estimated annual percentage change.

### Trends in migraine prevalence in China and globally

In 2021, the number of prevalent migraine cases in China was 184,752,280.11 (95% UI: 160,836,524–213,633,958.29), representing a 38.42% increase compared with 1990. Among these, male cases rose from 50,887,361.36 in 1990 to 70,036,222.95 in 2021, an increase of 37.63%, while female cases increased from 82,587,175.18 to 114,716,057.16, a 38.90% rise. The ASPR of migraine in China increased from 10,948.52 per 100,000 (95% UI: 9,428.76–12,586.13) in 1990 to 11,777.51 per 100,000 (95% UI: 10,137.56–13,538.56) in 2021, with an EAPC of 0.28 (95% CI: 0.24–0.33). The ASPR growth was slightly higher in males (EAPC: 0.30) than in females (EAPC: 0.27) (Table 1). In comparison, the global number of prevalent migraine cases in 2021 was 1,158,432,823.82 (95% UI: 995,861,966.38–1,331,312,506.13), reflecting a 58.13% increase since 1990. Globally, male cases increased from 269,667,853.33 in 1990 to 433,190,631.84 in 2021, a growth of 60.64%, while female cases rose from 462,896,609.35 to 725,242,191.97, an increase of 56.67%. The global ASPR also showed an upward trend, with an EAPC of 0.06 (95% CI: 0.05–0.08), and the increase was more pronounced in males (EAPC: 0.13) than in females (EAPC: 0.02) (Table 1, Figure 1).

### Trends in DALYs for migraine in China and globally

In 2021, the DALYs attributable to migraine in China were 6,988,198.60 (95% UI: 1,133,318.67–15,186,289.26), representing a 38.96% increase compared with 1990. Male DALYs increased from 1,987,263.27 (95% UI: 394,504.68–4,365,126.26) in 1990 to 2,745,320.72 (95% UI: 581,676.86–5,851,443.22) in 2021, a rise of 38.15%, while female DALYs increased from 3,041,524.19 (95% UI: 360,236.18–6,866,451.03) to 4,242,877.87 (95% UI: 548,619.60–9,335,915.43), a 39.50% increase. The ASDR in China rose from 412.97 per 100,000 (95% UI: 66.16–911.02) in 1990 to 443.65 per 100,000 (95% UI: 66.93–971.68) in 2021, with an EAPC of 0.27 (95% CI: 0.23–0.32), and the growth rates were similar between males and females (Table 1). In contrast, the global DALYs due to migraine in 2021 were 43,378,889.81 (95% UI: 6,732,642.21–95,079,454.09), representing a 58.25% increase since 1990. Male DALYs increased from 10,232,568.59 (95% UI: 1,675,317.81–22,688,369.40) in 1990 to 16,494,946.45 (95% UI: 2,802,071.63–35,551,602.46) in 2021, a growth of 61.20%, whereas female DALYs increased from 17,179,627.70 (95% UI: 2,400,079.70–37,859,718.47) to 26,883,943.36 (95% UI: 3,930,570.58–58,848,028.71), a 56.49% increase. Globally, the ASDR also exhibited an upward trend (EAPC: 0.05, 95% CI: 0.04–0.07), with a more pronounced increase in males (EAPC: 0.13) than in females (EAPC: 0.01) (Table 1, Figure 1).

### Analysis of age and gender differences in the burden of migraine in China and globally

**Figure 2:**
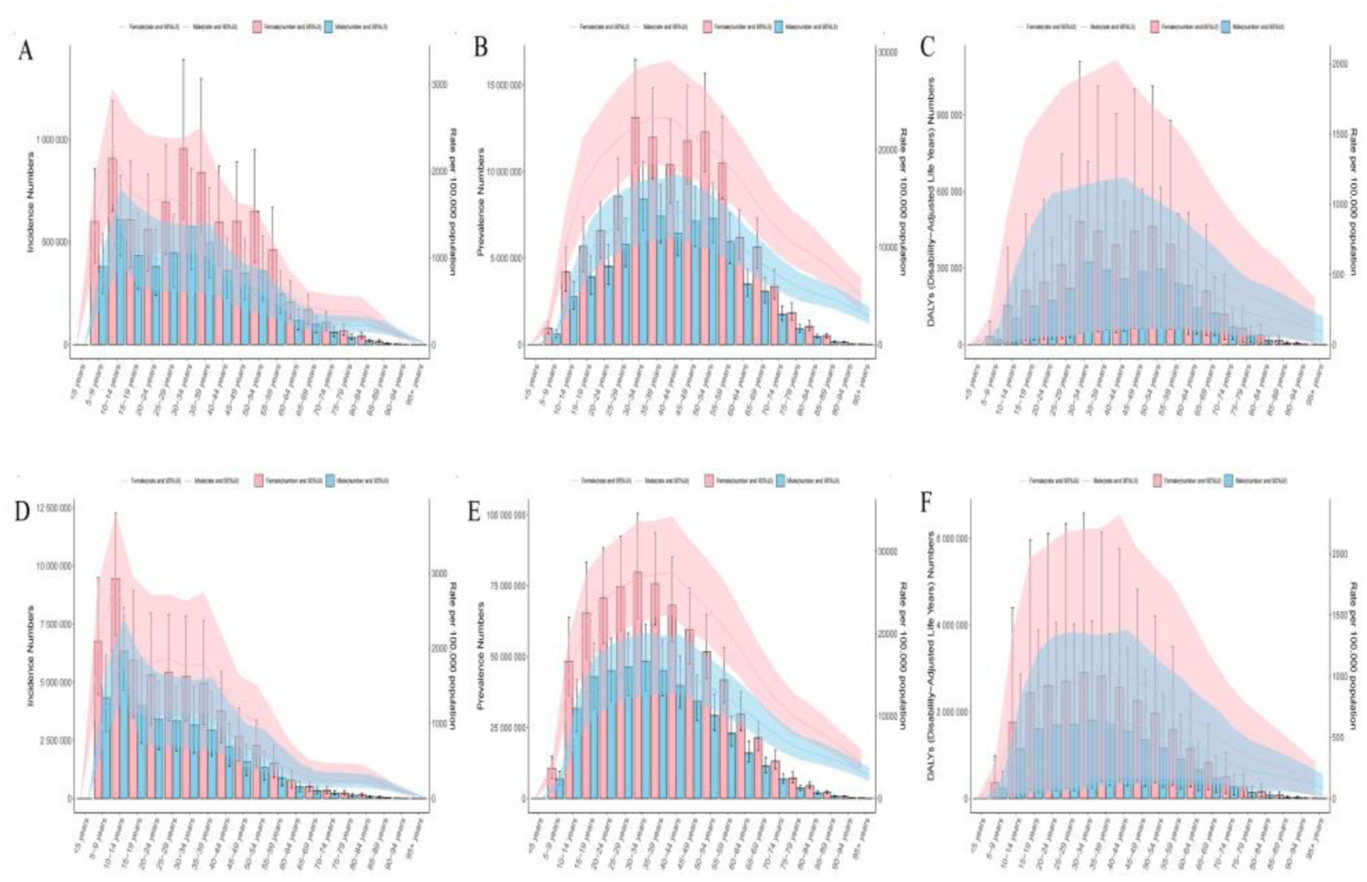
The number and rates of incidence, prevalence, and DALYs for migraine in specific age groups in China and globally in 2021, stratified by sex. **(A)** Number and rates of migraine incidence in specific age groups in China. **(B)** Number and rates of migraine prevalence in specific age groups in China. **(C)** Number and rates of migraine DALYs in specific age groups in China. **(D)** Number and rates of migraine incidence in specific age groups globally. **(E)** Number and rates of migraine prevalence in specific age groups globally. **(F)** Number and rates of migraine DALYs in specific age groups globally.

**Figure 3:**
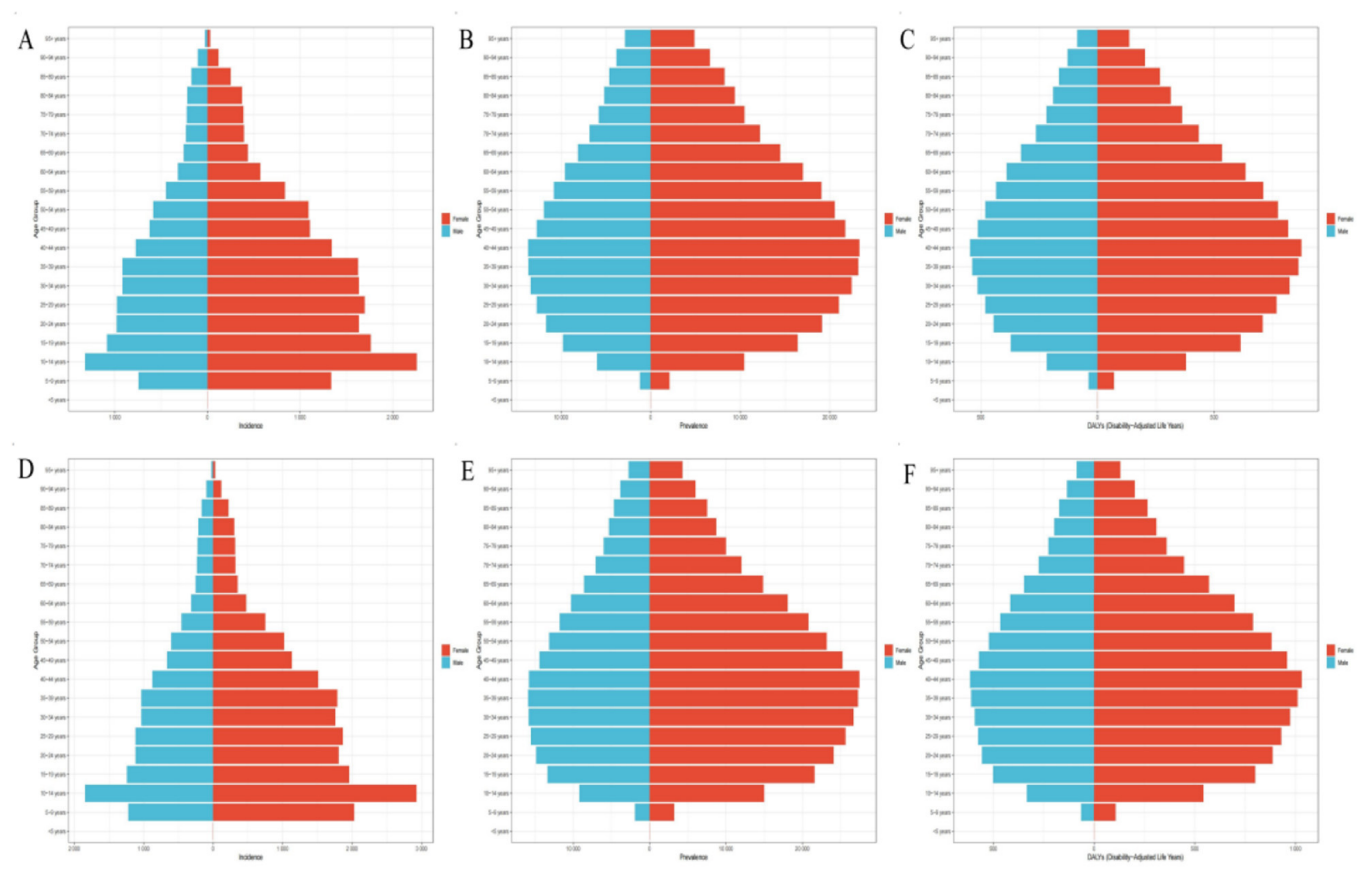
Comparison of the number of migraine incidence, prevalence, and DALYs in different age groups of males and females in China and globally in 2021. **(A)** Comparison of the number of migraine incidence in different age groups of males and females in China. **(B)** Comparison of the number of migraine prevalence in different age groups of males and females in China. **(C)** Comparison of the number of migraine DALYs in different age groups of males and females in China. **(D)** Comparison of the number of migraine incidence in different age groups of males and females globally. **(E)** Comparison of the number of migraine prevalence in different age groups of males and females globally. **(F)** Comparison of the number of migraine DALYs in different age groups of males and females globally.

In 2021, the peak number of migraine cases, prevalence, and DALYs in Chinese women occurred in the 30–34 age group. In contrast, the peak incidence of migraine cases in men was observed in the 10–14 age group, while the peak prevalence and DALYs were also observed in the 30–34 age group. Further analysis revealed that both the ASIR, ASPR, and ASDR for migraine in both females and males showed a trend of first increasing and then decreasing with age. Specifically, ASIR peaked in the 10–14 age group and then gradually declined, while ASPR and ASDR reached their maximum in the 40–44 age group and then began a gradual decline. Globally, both women and men showed a peak incidence of migraine in the 10–14 age group, with the peak prevalence and DALYs observed in the 30–34 age group, similar to the trend in China. The trends in ASIR, ASPR, and ASDR for women were similar to those in China, with all indicators rising and then falling with age, peaking at the 10–14, 40–44, and 40–44 age groups, respectively, before gradually declining. For men, the peak values of ASIR, ASPR, and ASDR occurred in the 10–14, 35–39, and 40–44 age groups, respectively, followed by a decline (Figure 2). It is noteworthy that, both in China and globally, all indicators related to migraine—such as incidence, prevalence, DALYs, ASIR, ASPR, and ASDR—are consistently higher in women than in men across all age groups, reflecting the unique risk that women face in terms of migraine burden. Moreover, the burden of migraine is concentrated mainly in the adolescent and young adult populations, with a more significant impact seen in women (Figure 3).

### Correlation analysis between the burden of migraine and socioeconomic development in China and globally

**Figure 4:**
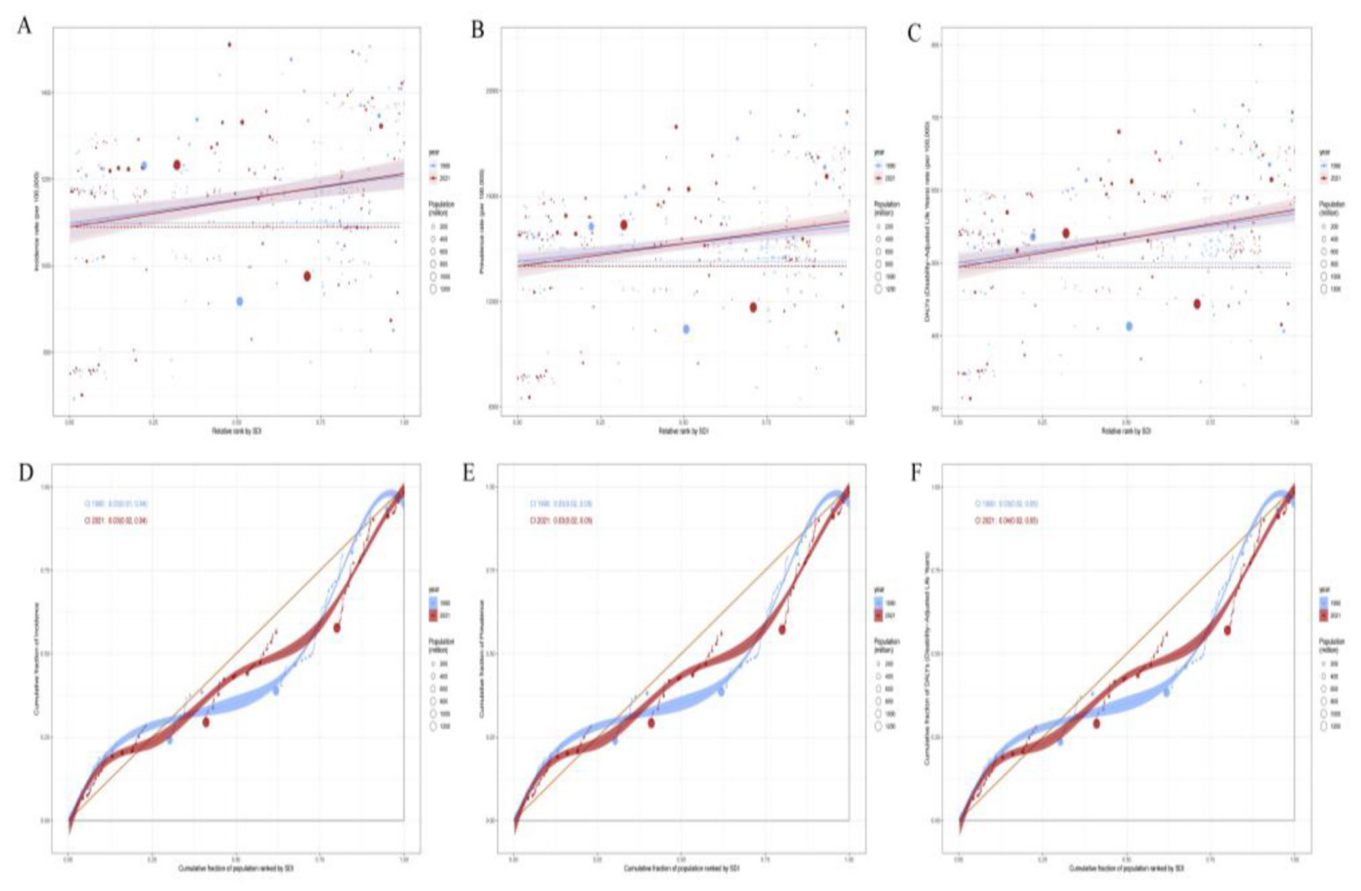
The health inequality regression curves and concentration curves for the burden of migraine across 204 countries and regions globally in 1990 and 2021. **(A–C)** Illustrate the slope index of inequality, describing the relationship between SDI and ASIR, ASPR, and ASDR, respectively, where each point represents an individual country or region weighted by population. **(D–F)** Display the concentration index, describing the relationship between SDI and ASIR, ASPR, and ASDR, respectively. This index quantifies relative inequality by integrating the area under the Lorenz curve. Blue represents data from 1990, and red represents data from 2021.

**Figure 5:**
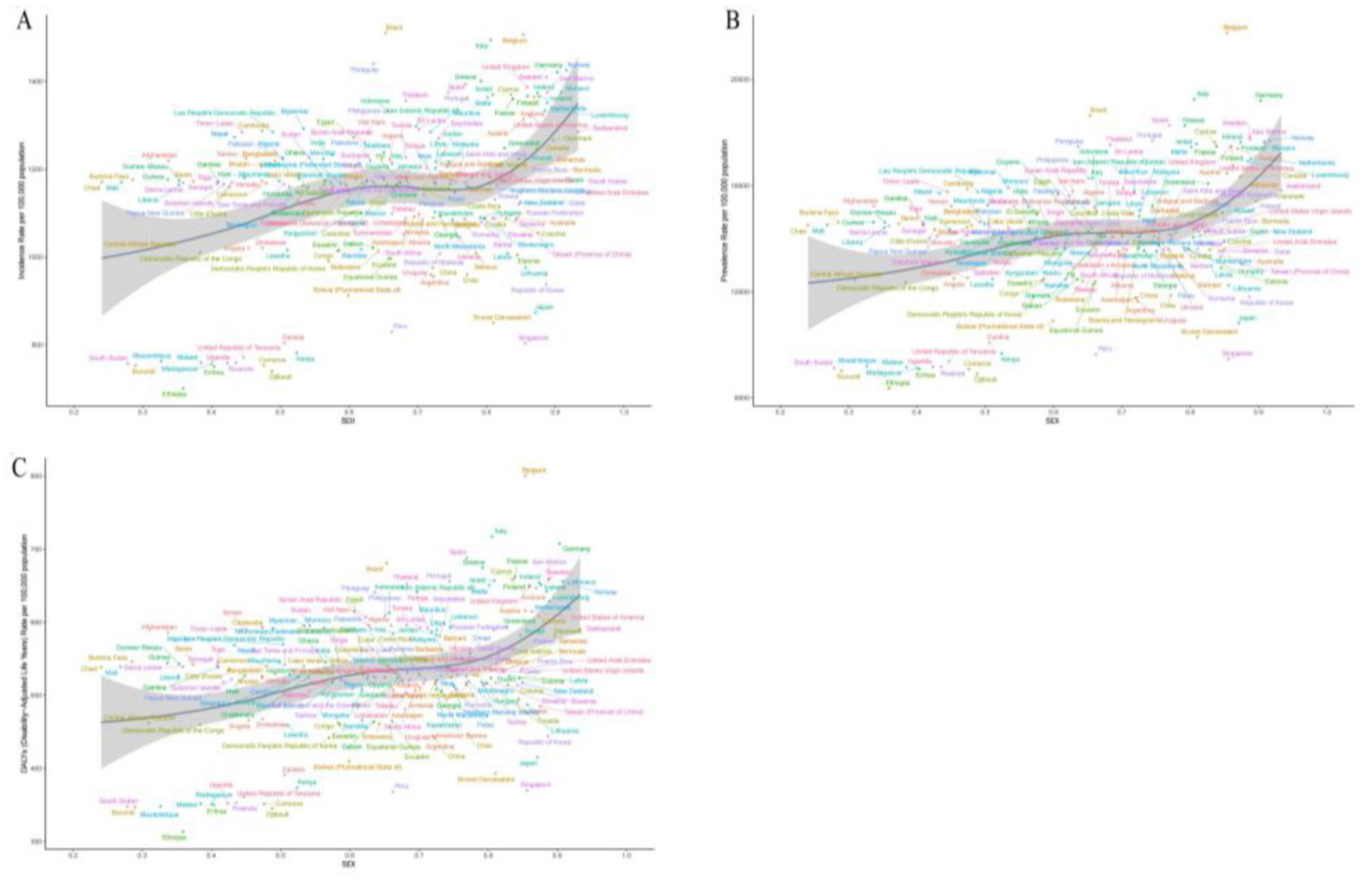
The relationship between the ASIR, ASPR, and ASDR of migraine and the SDI across 204 countries and territories globally from 1990 to 2021. **(A)** The relationship between the ASIR of migraine and the SDI across 204 countries and territories globally. **(B)** The relationship between the ASPR of migraine and the SDI across 204 countries and territories globally. **(C)** The relationship between the ASDR of migraine and the SDI across 204 countries and territories globally.

Health inequality analysis revealed notable disparities in migraine burden across the 204 countries and territories included in this study. The SII for ASIR increased from 110.00 per 100,000 (95% CI: 48.39–171.61) in 1990 to 123.21 per 100,000 (95% CI: 61.94–184.48) in 2021. Similarly, the SII for ASPR rose from 1,363.96 per 100,000 (95% CI: 550.94–2,176.97) to 1,705.26 per 100,000 (95% CI: 882.15–2,528.37), and the SII for ASDR increased from 67.75 per 100,000 (95% CI: 36.68–98.82) to 78.69 per 100,000 (95% CI: 48.71–108.68). These trends indicate that health inequalities in ASIR, ASPR, and ASDR exist globally, with the gap between the poorest and wealthiest countries gradually widening. Furthermore, the CII for ASIR, ASPR, and ASDR in both 1990 and 2021 were positive, suggesting that the migraine burden is predominantly concentrated in countries with higher socioeconomic development (Figure 4). In addition, from 1990 to 2021, ASIR, ASPR, and ASDR exhibited a positive correlation with the SDI, indicating that migraine burden tends to increase with higher levels of socioeconomic development (Figure 5).

### Decomposition analysis of the burden of migraine in China and globally

**Figure 6:**
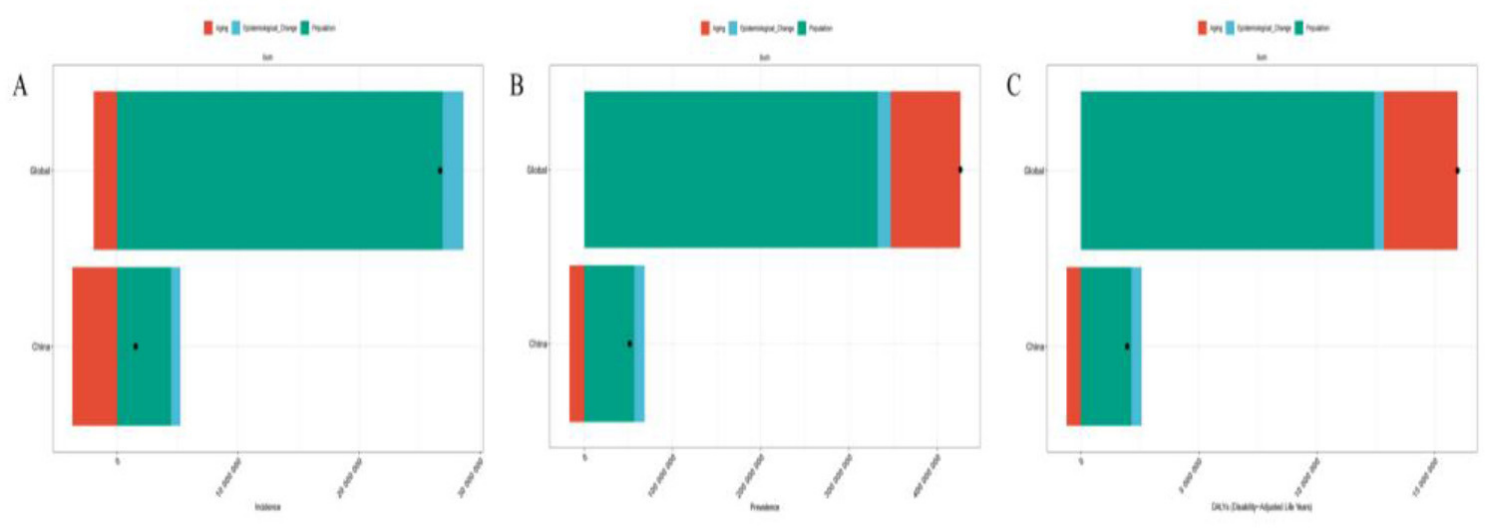
Decomposition analysis of migraine Incidence, Prevalence, and DALYs in China and globally from 1990 to 2021. **(A)** Decomposition analysis of migraine Incidence in China and globally. **(B)** Decomposition analysis of migraine Prevalence in China and globally. **(C)** Decomposition analysis of migraine DALYs in China and globally.

The decomposition analysis further elucidates the relative contributions of population growth, aging, and epidemiological changes to the burden of migraine in China and globally. Population growth emerged as the primary driver of the increasing incidence, prevalence, and DALYs of migraine in both China and worldwide, while epidemiological changes also played a role in shaping these trends. In contrast, aging contributed to a decline in migraine incidence in both China and globally and led to a reduction in migraine prevalence and DALYs in China. However, aging became a key factor driving the increase in migraine prevalence and DALYs globally, particularly in regions experiencing rapid aging. These findings highlight the distinct challenges faced by different regions in addressing the burden of migraine. When formulating targeted public health interventions, it is essential to comprehensively consider regional population structures, disease prevalence trends, and the varying impacts of aging to achieve precise and effective health management (Figure 6).

### Prediction analysis of migraine burden in China and globally

**Figure 7:**
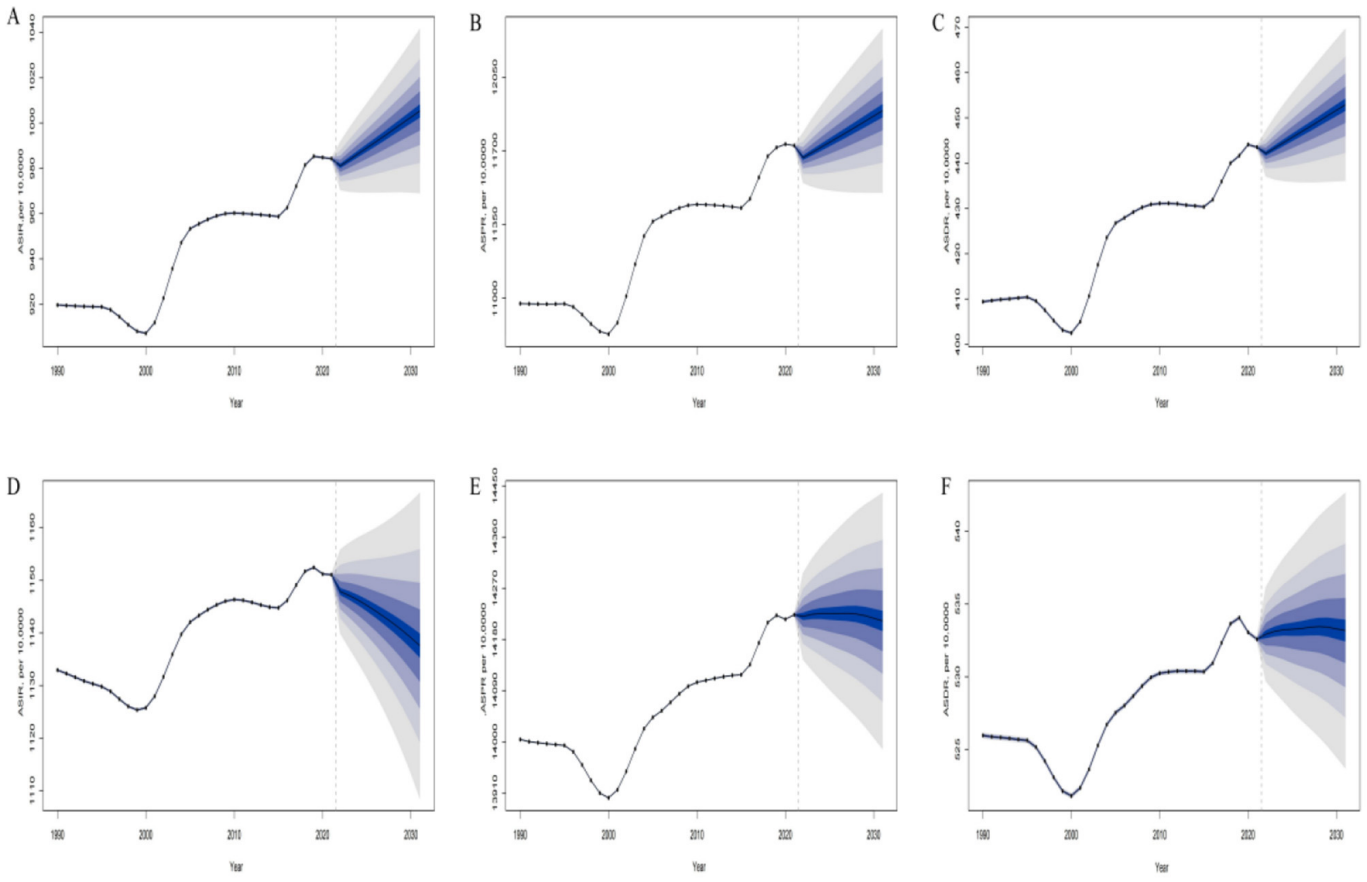
The BAPC model predictive analysis of ASIR, ASPR, and ASDR for migraine in China and globally from 2022 to 2031. **(A)** Predictive analysis of ASIR for migraine in China. **(B)** Predictive analysis of ASPR for migraine in China. **(C)** Predictive analysis of ASDR for migraine in China. **(D)** Predictive analysis of ASIR for migraine globally. **(E)** Predictive analysis of ASPR for migraine globally. **(F)** Predictive analysis of ASDR for migraine globally.

The prediction analysis indicates that, over the next decade, China's ASIR, ASPR, and ASDR for migraine are expected to continue rising. This trend may be closely linked to several factors, including population aging, lifestyle changes (such as increased stress, long working hours, irregular eating habits, etc.), and environmental pollution. More importantly, unique social and economic factors in China, such as accelerated urbanization and shifts in public health awareness, may also contribute to this trend to some extent. In contrast, the global trend for migraine's ASIR, ASPR, and ASDR is expected to show a slight decrease, likely attributed to continuous advancements in global public health, especially in developed countries. The widespread adoption of early screening technologies, improvements in treatment methods, and increased health awareness have gradually resulted in more effective early diagnosis and intervention for migraine. Furthermore, the global emphasis on chronic disease management and the implementation of integrated treatment strategies have also contributed to alleviating the migraine burden. However, the global decline trend is not uniform. Some low-income and middle-income countries, especially certain developing countries, still face a heavy migraine burden due to insufficient medical resources and health interventions, and the issue has not been effectively controlled. Therefore, while the global migraine burden has eased to some extent, the burden in China continues to rise. This requires greater attention at the policy and healthcare levels, as well as more personalized and refined intervention strategies to address the increasingly severe health challenges (Figure 7).

## Discussion

Based on data from the GBD 2021 study, this research systematically analyzed the trends and determinants of migraine burden in China and worldwide from 1990 to 2021. The findings indicate that although both China and the global population experienced an increasing burden of migraine, the magnitude of growth and the driving factors differed significantly. By 2021, the number of incident cases, prevalent cases, and DALYs attributable to migraine had all risen markedly compared with 1990 in both China and globally, with the global growth rate being substantially higher than that observed in China. However, during the same period, the increases in ASIR, ASPR, and ASDR were greater in China than at the global level, a divergence likely shaped by multiple interacting factors. First, the global increase in migraine burden was largely influenced by advances in healthcare systems in high-income countries. Improved capacity for early diagnosis and treatment in these regions contributed to the rise in cases and DALYs, but the relative adequacy of medical resources tempered the growth of age-standardized rates. In contrast, despite significant progress in healthcare investment and public health infrastructure in China—leading to improvements in diagnosis and treatment—several challenges remain. The large population base, uneven resource allocation, and limited healthcare accessibility in rural and remote areas constrain early detection and effective management of migraine, resulting in relatively greater increases in age-standardized rates (23–25). Second, lifestyle transitions have also played a critical role in driving the global burden of migraine. Accelerated urbanization, heightened psychosocial stress, and increasingly irregular lifestyles have contributed to rising migraine incidence, prevalence, and DALYs, particularly in high-income countries (26). China, undergoing rapid urbanization and socioeconomic transformation, faces similar challenges. However, differences in population structure and epidemiological transitions may explain why the increases in age-standardized rates have been more pronounced in China than globally.

This study further highlights the importance of gender differences in the burden of migraine. Women consistently accounted for the majority of the migraine burden, particularly during periods of physiological change such as puberty, pregnancy, and menopause, where fluctuations in estrogen provide the biological basis for the heavier burden observed in females (27–29). In addition, social stressors, family responsibilities, and workplace demands may further exacerbate the burden of migraine among women, especially in the context of modern society's expectations for women to assume multiple roles (30). Nevertheless, the burden of migraine among men has also shown an upward trend in recent years, particularly among younger males. Prolonged screen exposure, increased occupational stress, and irregular sleep patterns may be the main contributors to this increase. These findings suggest that although women remain disproportionately affected by migraine, the rising burden among men warrants greater attention and should be effectively addressed in future public health interventions. Furthermore, the highest burden of migraine was observed among adolescents and young adults, whose quality of life is particularly vulnerable. Given the high levels of productivity and social engagement in this age group, migraine imposes a disproportionately negative impact on their daily life, work, and academic performance.

Health inequality analysis revealed that the global burden of migraine is closely associated with the level of socioeconomic development. Countries with a high SDI bear a heavier migraine burden, and the burden tends to increase with rising socioeconomic levels. This trend is largely attributed to well-established healthcare infrastructure, widespread access to medical services, and improved rates of early diagnosis in these countries. Although advanced public health systems in high-SDI countries facilitate early recognition and treatment of migraine, changes in modern lifestyle, occupational and social stress, particularly psychological strain and environmental pollution, continue to exacerbate the disease burden. Prolonged screen exposure, intensified occupational competition, and work–life imbalance have emerged as key drivers of the rising migraine burden (31, 32). In contrast, low-SDI countries generally report a lower migraine burden; however, the actual burden is often underestimated due to limited healthcare resources, inadequate diagnostic capacity, and insufficient health education, which hinder widespread diagnosis and treatment. Underdeveloped healthcare systems, along with a shortage of facilities and trained professionals, prevent timely management of migraine, while the prevalence and socioeconomic impact of the disease remain insufficiently quantified.

Decomposition analysis further revealed the relative contributions of population aging, population growth, and epidemiological changes to the trends in migraine burden in China and globally. The findings indicated that population growth was the primary driver of the increases in incidence, prevalence, and DALYs of migraine both in China and worldwide. In particular, in high-SDI countries and China, the overall expansion of the population directly contributed to the rising number of migraine cases. Epidemiological changes also partly influenced these indicators. However, the impact of aging varied across regions. In China, population aging had a relatively minor effect on incidence, whereas younger populations, especially females, exhibited a higher prevalence. Lifestyle changes and social pressures may have further aggravated the burden in this demographic group. By contrast, at the global level, particularly in regions experiencing rapid population aging, demographic shifts substantially exacerbated the migraine burden. In high-income countries, the prevalence and DALYs of migraine among older adults increased markedly, making aging a critical factor driving the global rise in migraine burden. These results underscore the regional heterogeneity in challenges associated with addressing migraine burden.

Forecasts indicate that the burden of migraine in China will continue to rise over the next decade, particularly among young adults and women, driven by socioeconomic transitions, lifestyle changes, and population aging. In contrast, the overall global growth rate of migraine burden is expected to slow, although low- and middle-income countries may still experience an upward trajectory, reflecting structural disparities such as limited healthcare resources and inadequate health management. Addressing this complex challenge requires a comprehensive, multi-level prevention and control strategy. First, it is essential to strengthen primary healthcare capacity to improve early recognition and timely intervention for migraine. Second, health education and lifestyle interventions should be promoted to enhance public awareness of the disease and its triggers, particularly among high-risk populations. Third, the advancement of precision medicine and individualized treatment, informed by genomic and neuroscience research, should be leveraged to develop tailored management strategies. Fourth, global public health collaboration is needed to facilitate technology transfer and the equitable distribution of medical resources, thereby narrowing the gaps in diagnosis and treatment across regions. Finally, an interdisciplinary approach that integrates psychology, sociology, and medicine should be encouraged to optimize migraine management in a systemic manner, ultimately improving patients' quality of life while alleviating the associated social and economic burden.

Meanwhile, optimizing disease assessment and treatment paradigms is equally critical for reducing the long-term burden of migraine. Patient-reported outcome measures (PROMs) have been widely applied in the field of headache to evaluate functional impairment and quality-of-life impacts (33). However, limitations remain in terms of sensitivity, cross-cultural comparability, and the ability to capture long-term outcomes, underscoring the need for further refinement and standardization. On the therapeutic side, recent large-scale studies highlight that the application pattern of anti-CGRP therapies requires a paradigm shift (34). Rather than being restricted to a “last-resort” option following prior treatment failure, these agents should be integrated earlier into the treatment framework to maximize clinical benefits and mitigate the socioeconomic burden of migraine. While this study, based on the GBD 2021 database, provides a systematic analysis of migraine burden trends in China and worldwide, it is subject to several limitations, including incomplete data coverage, regional variability in diagnostic criteria, and uncertainties inherent in the assumptions of predictive models. Future research should focus on dynamic data collection, methodological improvement of PROMs, and rigorous evaluation of emerging therapeutic strategies to achieve more precise, sustainable prevention and management of migraine.

## Conclusions

This study systematically analyzed the trends in migraine burden in China and globally from 1990 to 2021, revealing the multidimensional influences of gender differences, socioeconomic factors, population growth, and modern lifestyle changes. The increasing global burden of migraine is closely linked to medical advancements in high-income countries, whereas in low- and middle-income countries, the burden may continue to rise due to insufficient healthcare resources. Future migraine prevention and control strategies should focus on low- and middle-income countries, promoting precision medicine, improving primary healthcare capacity, and strengthening health education and personalized treatment approaches. Particular attention should be given to young populations, women, and high-risk groups by enhancing public health interventions, optimizing lifestyle habits, and reducing the impact of psychological stress and environmental pollution. This study provides a crucial perspective on global migraine burden trends and aims to offer theoretical guidance for future public health policies and prevention strategies.

## Data Availability

The datasets presented in this study can be found in online repositories. The names of the repository/repositories and accession number(s) can be found below: https://vizhub.healthdata.org/gbd-results/.
